# The Consumption of Milk or Dairy Products and Sleep Quality: A Systematic Review and Meta-Analysis

**DOI:** 10.7759/cureus.92556

**Published:** 2025-09-17

**Authors:** Andrea Pissatto Peres, Yasmin Ourives Domingues, Bruna Teles Soares Beserra, Nathalia Sernizon Guimarães, Juliana Aparecida Correia Bento, Yuri Duarte Porto, Bruno Serpa Vieira, Maressa Caldeira Morzelle

**Affiliations:** 1 Department of Food and Nutrition, Federal University of Mato Grosso, Cuiabá, BRA; 2 Department of Nutrition, Nursing School, Federal University of Minas Gerais, Belo Horizonte, BRA; 3 Faculty of Veterinary Medicine and Zootechnics, Federal University of Uberlândia, Uberlândia, BRA

**Keywords:** insomnia, psqi test, sleep duration, sleep latency, yogurt

## Abstract

Sleep quality is a key component of overall health and well-being. Despite its importance, many individuals experience sleep disturbances and seek various strategies to improve their sleep. The consumption of milk and dairy products has been linked to mood regulation and may influence sleep quality. However, the scientific evidence remains inconsistent, with studies reporting mixed and inconclusive results. The aim of the study is to investigate whether consuming milk and dairy improves sleep quality. A systematic review and meta-analysis were conducted using electronic databases PubMed, Science Direct, Embase, and Web of Knowledge. We included randomized controlled trials (RCTs) that evaluated the effects of the consumption of milk and dairy products on sleep quality. The meta-analysis was conducted using a random-effects model, calculating mean differences (MD) using Review Manager 5.4.1 and Jamovi 2.3.19.0. A total of 2,398 records were screened, and nine clinical trial studies were selected for the qualitative synthesis (systematic review). Four clinical trials met the criteria for inclusion in the meta-analysis following a thorough literature review. Meta-analysis results from the findings of clinical trials demonstrated that milk and dairy product consumption significantly reduced the Pittsburgh Sleep Quality Index (PSQI) total score (p < 0.01), regardless of treatment conditions or study groups, suggesting an overall improvement in sleep quality. The findings suggest that milk and dairy product consumption may contribute to improved sleep quality, as evidenced by self-reported measures in clinical trials. Further high-quality studies in humans are needed to confirm these effects.

## Introduction and background

The quality of sleep is affected by several factors, including lifestyle habits, environmental conditions, and psychological states [1]. Sleep quality plays a crucial role in overall health and well-being. Good quality and sufficient quantity of sleep are essential for optimal cognitive performance, academic achievement, and for preventing health issues and psychiatric disorders [2]. However, many individuals have sleep problems and search for various methods to improve their sleep quality. Currently, there are several treatment options available, including both non-pharmacological and pharmacological approaches. These include the use of melatonin, antipsychotic medication, adrenergic agonists, antidepressants, and sedative-hypnotic drugs [3]. Previous research has highlighted the complex and bidirectional nature of the relationship between diet and sleep quality. A substantial body of evidence from both population-based and animal studies indicates a beneficial association between milk consumption and sleep disorders [4,5].

The association between milk consumption and sleep quality has been a topic of interest in recent years, leading to an increasing number of studies in this area [6]. Therefore, the relationship between milk consumption and sleep quality has been investigated in several studies [7-13]. Some studies have suggested that milk and dairy, particularly milk rich in melatonin and tryptophan, may have sleep-promoting effects [14].

In terms of mechanism of action, the substantial presence of tryptophan (Try) in milk and dairy, from which melatonin is synthesized, may exert a suppressive effect on the activity of the inhibitory neurotransmitter gamma-aminobutyric acid (GABA) [6]. Based on the findings of these studies by animal models, cow milk, dairy, and GABA-rich fermented milk improved sleep duration in insomnia-induced mice [8,14-17].

One study conducted in Finland found that milk harvested at night, which contained high levels of melatonin, showed promising sleep-promoting effects in humans [18]. In addition, many studies in humans and rats point to a warning about supplementing with melatonin, as it may not be as well absorbed as the melatonin present in greater quantities in milk from night milking or the melatonin produced by the mitochondria [19].

However, other studies by clinical trials have found no significant effect of milk consumption on sleep quality [10,13]. Komada, Okajima, and Kuwata [6] performed a systematic review to summarize the literature and provide a unified view on the intake of milk and dairy. As a result, a total of 14 studies published between 1972 and 2019 were included in this review, including eight randomized controlled trials, two experimental studies with crossover designs, one longitudinal study, and three cross-sectional studies. Four studies targeted older adults, three included toddlers, two targeted children, and six enrolled adults, including university students. Overall, these studies indicated that a well-balanced diet that includes milk and dairy products is effective in improving sleep quality, despite mixed results across studies attributable to differences in study populations and methods [6]. However, the authors did not conduct a meta-analysis.

Given the biological plausibility, supported by the presence of sleep-regulating compounds such as melatonin and tryptophan in milk and dairy products, and the mixed findings from both human and animal studies, we hypothesize that the consumption of milk and dairy products may have a beneficial effect on sleep quality. In light of the conflicting results reported in the literature, a systematic review and meta-analysis are warranted to synthesize the existing evidence and clarify the overall impact of milk and dairy intake on sleep quality. This review aims to critically evaluate and quantify the effects of milk and dairy product consumption on sleep parameters based on the available scientific literature. In summary, the study aims to investigate whether consuming milk and dairy improves sleep quality.

## Review

Material and methods

Statement and Registration

This systematic review and meta-analysis followed the recommendations of the Preferred Reporting Items for Systematic reviews and Meta-Analysis (PRISMA) [20] and was registered on PROSPERO (CRD42024523674).

Search Strategy

The literature search strategy was developed using the PICOS strategy, in which P (population) was defined as ‘human adults and animal models,’ I (intervention) as ‘consumption of milk or dairy products,’ C (comparator), as in ‘comparison control group without consumption of milk or dairy products,’ O (outcome) as ‘sleep quality/latency time and sleep duration/sleep efficiency. We searched PubMed, Scopus, Web of Science, and Embase from inception to May 30, 2025. The search strategy used was the following: (“milk” OR "cow milk" OR "dairy product" OR "cultured milk product" OR “yogurt” OR “cheese” OR "fermented milk products" OR “butter” OR “buttermilk”) AND (“sleep”). No other filter to refine the search was used, no language restrictions were applied, and the period was since 1989, when the PSQI started to be evaluated. We also manually searched the reference lists of included studies and related reviews.

Eligibility Criteria, Study Selection, and Data Extraction

Two independent reviewers (APP and YOD) selected the studies in two phases. First, titles and abstracts were screened from the retrieved articles to determine eligible studies. In the second phase, the full-text version of eligible studies was read by both reviewers independently to apply the inclusion criteria. Disagreements were resolved through discussion with a third reviewer (MCM). We included studies of interventions addressing the effect of milk and dairy product consumption on sleep quality in adult humans (up to 18 years). Therefore, clinical trial studies with PSQI outcomes. We excluded studies involving anamnesis and without intervention that used non-cow's milk and that did not evaluate sleep quality. Furthermore, abstracts, reviews, editorials, case reports, case series, study protocols, pilot studies, animal models, and in vitro studies were also excluded. The following information was collected from the included studies: author, year of publication, country, type of study, population (age and size), intervention (dose and duration of intervention), dependent variable, outcomes, and main findings.

Risk of Bias

The risk of bias was assessed independently by two reviewers (APP and JACB) using the tool RoB 2.0 for randomized studies [21], as can be seen in Table 1. Disagreements were resolved by discussion with a third reviewer (MCM). Additionally, we provide a Heyland Methodologic Quality Score for studies included in qualitative synthesis [22,23].

**Figure 1:**
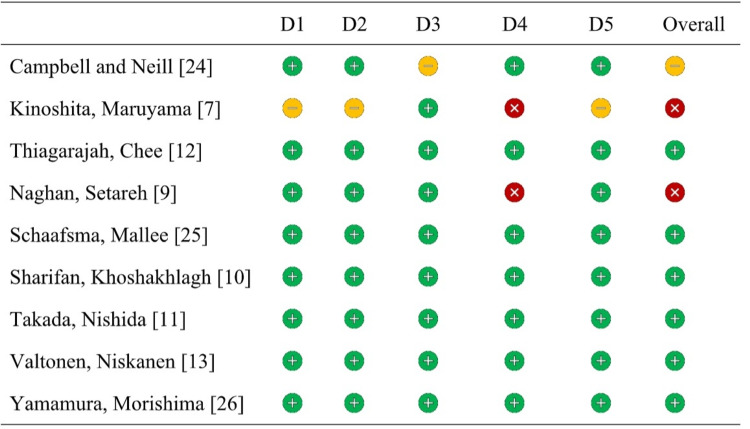
Risk of bias was assessed by using RoB 2.0 for randomized studies (studies by clinical trial).

The risk of bias of studies included in the systematic review and meta-analysis was assessed using five domains of bias arising from the randomization intervention, intended intervention, missing outcome data, measurement of the outcome, and bias in selection of the report result. Two studies included in this review were classified as high overall risk, one study as some concerns, and the others were classified as being of low risk of bias (Figure 1).

Assessment of Quality

The overall quality of the studies included in the systematic review and meta-analysis was assessed by applying the above criteria regarding randomization, analysis, blinding, patient selection, comparability of groups at baseline, extent of follow-up, treatment protocol, cointerventions, and outcomes. All of the studies were ranking more than eight points and are being assessed as high quality [23].

Meta-Analysis

The measure of the effect size of each variable was the mean difference (MD) between the comparison, control (non-milk group), and intervention (milk intake group) groups, as follows:

MD = mean of intake milk or dairy product (DP) group - mean of control (non-milk) group

The studies were weighted using the inverse of variance method to balance their individual contributions to the meta-analysis based on their level of precision in estimating the treatment means. The significance of the overall mean difference (overall effect) was obtained by the Z test (p<0.05). Moreover, 95% confidence intervals were calculated for each observation.

Between studies, the heterogeneity was checked by the chi-square test (X2) (p<0.10), and its magnitude was estimated by the inconsistency index (I2 = Ch2 - DF / Ch2 x 100), where DF is the degrees of freedom of the Ch2 test. Regardless of its significance, the heterogeneity between studies was incorporated into the meta-analysis, adopting a random effects model to assess overall effects and their statistical significance. In the case of significant heterogeneity between studies, meta-regression and subgroup analyses were performed to explain at least part of its origin, considering the variations between studies in terms of product type, dose intake, and method of analysis. In these cases, quantitative data were grouped into ranges of values to facilitate their incorporation into the analysis.

The meta-analysis results' robustness was confirmed through a sensitivity analysis, which included plotting each RD against its corresponding standard error (funnel plot). The absence of publication bias was established by a homogeneous distribution of RDs on both sides of the funnel plot. Also, funnel plot asymmetry was formally tested using Egger's regression. Studies with data outside the normality area of the funnel plot were excluded from the meta-analysis. The 'fill and trim' method was used to reintroduce them into the database only if their exclusion did not significantly interfere with the estimation of the effect size and the value of the general test. All statistical analysis and graphical synthesis of results were performed using Review Manager 5.4.1 and Jamovi 2.3.19.0 software.

Results

Study Selection

**Figure 2:**
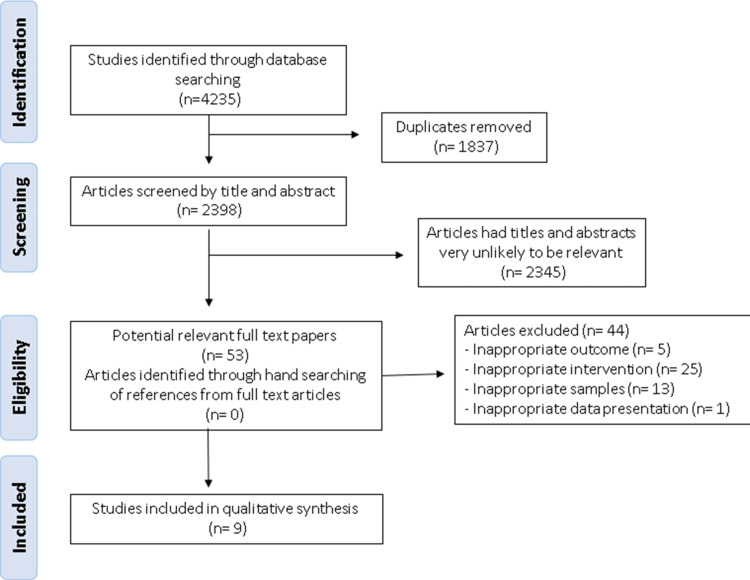
Flow diagram of study selection for systematic review following the preferred reporting items for systematic reviews and meta-analyses (PRISMA) guidelines. [20]

We identified and screened 4,235 records, reviewed 53 full texts for eligibility, and included nine studies by clinical trial in the review: Campbell and Neill [24]; Kinoshita, Maruyama [7]; Thiagarajah, Chee [12]; Naghan, Setareh [9]; Schaafsma, Mallee [25]; Sharifan, Khoshakhlagh [10]; Takada, Nishida [11]; Valtonen, Niskanen [13]; Yamamura, Morishima [26]. Figure 2 presents a flow diagram illustrating the selection process.

In the meta-analysis [27-29], articles were excluded due to the lack of the Pittsburgh Sleep Quality Index (PSQI) method to assess sleep quality in humans (n=5). Besides that, these excluded articles were used to compare sleep quality between humans and animals for all the methods found. The main reasons for excluding studies from the systematic review were: (a) studies of surveys or reviews without intervention; (b) studies with no outcome for sleep; and (c) studies where samples were not of milk or dairy products.

Description of Included Studies

The sleep quality tested in selected articles varied among fermented milk [11,26], night and day milk [13,24], yogurt [7,9,10], low-fat milk with supplementation [12], and whey-protein galacto-oligosaccharides-based product (DP) [25]. The main results of the studies included in the review on the effect of milk and dairy products on sleep quality in humans are summarized in Table 1.

**Table 1:** Primary findings of the studies included in the systematic review that examined the impact of milk and dairy product consumption on human sleep quality.

Author, Year, country	Study design	Population	Intervention	Treatment	Dependent variable	Main results
Campbell and Neill [24] New Zealand	Randomized double blind placebo-controlled crossover trial over 9 weeks	Adults with primary insomnia (mean age 40 ± 14 years) (n = 19)	Product A: night-milk (melatonin 85.5 pg/ml) modified with an alpha s1-ca-sein tryptic hydrolysate	250 mL a day 30 min before bedtime for 63 days	PSQI questionnaire, ISI, Polysomnography and Actigraphy	- Consumption of NightMilk significantly improved sleep efficiency by actigraphy compared to placebo. - NightMilk significantly reduced the time taken to fall asleep compared to placebo. - NightMilk significantly improved subjective sleep quality scores compared to placebo. - NightMilk significantly improved the total sleep quality score over one week compared to placebo.
			Product B: day-milk			
Kinoshita, Maruyama [7] Japan	Randomized, controlled, and open-label design	Women healthcare workers, 20–71 years (n = 961)	Control group (did not consume yogurt during this period)	112 mL of L. delbrueckii yogurt daily for 112 days	PSQI questionnaire, SF-8, and GSRS questionnaire	- Participants in the yogurt group had a statistically significant improvement in PSQI scores compared to the control group. - The intervention effects were significant in the General Health subscale and Vitality subscale scores of SF-8. - The study had several limitations, including the lack of blinding and the use of an open-label design.
			Yogurt fermented with Lactobacillus delbrueckii ssp. bulgaricus			
Thiagarajah, Chee [12] Malaysia	A Double Blind, Randomized Placebo-Controlled Crossover Trial	Academic staff over 18 years old (n=39)	Placebo capsules (low-fat milk powder)	1 capsule daily for 28 days	PSQI questionnaire, salivary cortisol by high-performance liquid chromatography method and alpha power of awake EEG	- Both the supplement and placebo groups showed improvements in sleep quality, as measured by the PSQI. - The supplement group showed greater improvements in several PSQI components, including sleep latency, sleep duration, and sleep efficiency. - The supplemented group also showed significant reductions in cortisol levels, which are associated with stress and poor sleep quality. - No significant adverse effects were reported in either group.
			Supplement capsules (150 mg Alpha-s1-casein tryptic hydrolysate and 50 mg L-theanine)			
Naghan, Setareh [9] Iran	Clinical trial with random sequences	Healthy Young Adults, 20-40 years (n = 17)	Placebo (water)	250 mL daily for 20 days	Test of variables of attention and sleepiness test via the visual analogue scale	- Doogh caused sleepiness in the afternoon time along with a significant increase in reaction time and commission and omission errors. - The timing and activity type of the drinker should be taken into account when considering the cognitive effects of Doogh.
			Doogh (a traditional dairy product (yogurt) in Iran)			
Schaafsma, Mallee [25] Netherlands	Randomized Controlled Cross-Over Study	Adults with sleep disturbances (PSQI>9), 30-50 years (n = 70)	Placebo (skimmed milk powder)	150 ml consumed once daily for 42 days	PSQI for sleep quality, saliva samples for cortisol evaluation and fecal samples for gut microbiota evaluation.	- The study found that the whey-protein and galacto-oligosaccharides based product had a positive effect on sleep quality and gut microbiota composition. - The study also found a trend towards a reduction in stress levels with the use of the product.
			Whey-protein galacto-oligosaccharides based product			
Sharifan, Khoshakhlagh [10] Iran	Parallel blind randomized controlled clinical trial	Adults with abdominal obesity, 30–50 years (n = 289)	Milk control	200 ml/day for 70 days	ISI questionnaire and quality of life was assessed using the Short Form Health Survey - SF-36 questionnaire	- Low-fat milk and yogurt fortified with 1,500 IU vitamin D3 can reduce insomnia symptoms and improve quality of life in individuals with serum vitamin D levels <30 ng/ml. - There were no significant differences observed in the control groups who received non-fortified milk or yogurt. - Serum 25(OH)D levels significantly increased in the intervention groups after the 10-week trial. - The study suggests that vitamin D3 fortification of dairy products may be an effective strategy to improve vitamin D status and reduce insomnia symptoms in populations with low vitamin D levels.
			Milk intervention (fortified with vit. D3)			
			Yogurt control	150 g/day of yogurt for 70 days		
			Yogurt intervention (fortified with vit. D3)			
Takada, Nishida [11] Japan	A double-blind, placebo- controlled trial	4th year medical students (n = 94)	Placebo (milk)	100 ml of LcS-fermented milk or non-fermented placebo milk for 77 days	Subjective anxiety, overnight single-channel EEG recordings, and the OSA sleep inventory scores of subjective sleep quality.	- Daily consumption of LcS fermented milk beverage improved sleep quality in healthy medical students exposed to academic stress. - LcS prevented the onset of abdominal dysfunctions and cold symptoms in healthy subjects exposed to academic stress. - LcS attenuated a stress-induced rise in salivary cortisol. - LcS preserved the diversity of gut microbiota.
			Lactobacillus casei strain Shirota fermented milk			
Valtonen, Niskanen [13] Finland	Double-blind, placebo-controlled study	Elderly institutionalized (n = 151)	Normal milk (day-milk)	0.61 L of milk /person/day for 56 days	The sleep quality using the ‘‘Mini-Mental state’’ scale	- The study found that there was no significant difference in sleep quality between night milk and normal milk periods for Group I, but for Group II, which consumed night milk during Period 2, sleep quality decreased during the later period. - The seasonal effect was statistically significant, covering all other possible effects. - The study found that there was no significant difference in sleep quality between subjects with severe and mild dementia. - The study did not find any significant side effects or risks associated with melatonin supplementation for elderly individuals.
			Night-time milk (10-40 ng/l melatonin)			
Yamamura, Morishima [26] Japan	Randomized, double-blind and placebo-controlled	Healthy elderly, 60–81 years (n = 29)	Placebo drink (artificially acidified milk)	100 g per day for 63 days	Sleep quality by means of actigraphy and quality of life by Short Form Health Survey - SF-36	- There was a significant improvement in sleep efficiency (P¼0.03) and number of wakening episodes (P¼0.007) in actigraph data after intake of fermented milk, whereas no significant changes were observed for the placebo. - Fermented milk did not improve the SF-36 scores significantly from the baseline period. In the general health domain of the SF-36, however, there was marginal improvement as compared to the baseline period. - Although the difference between fermented milk and placebo was not statistically significant for any of the sleep or Quality of life parameters, fermented milk produced slightly greater mean values for many parameters.
			Fermented milk drink			

Meta-Analysis

A total of nine articles were selected for the meta-analysis, while five studies by human trial were excluded because they did not present PSQI data.

The variables sleep efficiency and sleep latency were not described in this study for clinical trials because they were only found in two selected articles, which did not meet the minimum requirements for evaluation in the meta-analysis. On the other hand, the PSQI total score was submitted to the meta-analysis based on its level of precision in estimating the treatment means.

**Figure 3:**
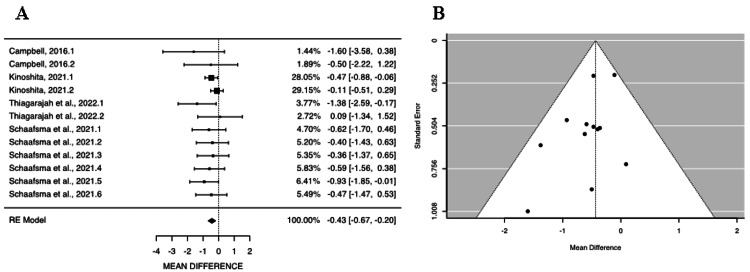
Effect of milk and dairy product consumption on sleep quality as measured by the Pittsburgh Sleep Quality Index (PSQI) in clinical trials. (A) Forest plot showing the standardized mean difference (SMD) in PSQI total scores data (n = 4 articles, k = 12 treatments) (I² = 4.8708%, p = 0.7030), (B) Funnel plot assessing publication bias among clinical trials. [27,7,12,28]

The estimated average mean difference based on the random-effects model for PSQI total score was -0.4349 (95% CI: -0.6729 to -0.1970), indicating a significant difference from zero (z = -3.5828, p = 0.0003) (Figure 3A). The 95% prediction interval for the true outcomes is -0.7380 to -0.1319. These results indicate that the true outcomes of the studies are generally aligned with the estimated average outcome. Based on the Q-test, there was no significant heterogeneity in the true outcomes (tau² = 0.0092, p = 0.7030), indicating that the milk and dairy products reduced the PSQI total score independent of the treatment conditions or groups studied, meaning that milk and dairy products improved sleep quality. An examination of the studentized residuals reveals that none of the studies have a value larger than ± 2.8653, indicating the absence of outliers in the context of this model (Figure 3B). Moreover, the Egger’s regression test indicates no funnel plot asymmetry (p = 0.1284).

Discussion

Based on the meta-analysis, it can be concluded that the consumption of dairy products is associated with an increase in sleep duration by 0.669 h (40.14 minutes). Furthermore, the consumption of fresh milk is linked to an increase in sleep duration of 0.160 h (9.6 minutes) (Table 2). In this way, we confirm the hypothesis that the consumption of milk and dairy products has a positive influence on sleep quality.

**Table 2:** Metaregression analysis was conducted to confidently identify the factors that may be responsible for the observed heterogeneity in sleep duration and sleep latency.

Moderator		Sleep Duration		
	k	Effects (95% CI)	p-value	R2 (%)
Product	15	-	0.016	27.6
Milk	7	0.160 (0.018 – 0.301)	0.028	-
Dairy	8	0.669 (0.249 – 1.089)	0.002	-
Intervention duration	15	-	0.017	33.04
> 7 days	10	0.206 (0.041 – 0.372)	0.015	-
< 15 days	2	-	-	-
Not informed	3	0.743 (0.162 – 1.324)	0.012	-
Moderator		Sleep latency		
	k	Effects (95% CI)	p-value	R2 (%)
Product	8	-	0.423	0
Dose	8	-	0.423	0
Intervention duration	8	-	0.001	97.29
> 7 days	6	-5.210 (-5.884 – -4.536)	0.001	-
< 15 days	2	-	-	-

Another subgroup that explains part of the observed heterogeneity for sleep duration was the treatment time (Table 2), where both evaluated treatment periods (less than seven days or greater than 15 days) presented a positive estimated mean. Thus, it is assumed that longer treatment times with milk and dairy products increased sleep duration in all animal trial studies evaluated.

Milk and dairy products contain a variety of minor compounds that have health benefits, such as bioactive peptides and melatonin, for their ability to promote and facilitate sleep [30]. Bioactive peptides can be derived from the casein or whey fraction of milk and are released in vivo because of proteolytic activity in the stomach and/or intestines. The sequence of milk protein peptides is capable of interfering with various biological functions, from hormone secretion, immune function, nutrient absorption, and transmission of neurological information to microbial growth [31]. Previous research has also reported the anxiolytic and sedative effects of milk and its constituents, such as S1-casein tryptic hydrolysate and lactopeptides, which have been shown to possess sedative and anxiolytic properties. These findings suggest that milk consumption may have a positive impact on sleep quality by promoting relaxation and reducing anxiety [17], which supports the results obtained by the meta-analysis. Moreover, milk and dairy product consumption improves mental functioning and increases physical functionality during the day, which may contribute to improved sleep quality [6].

Milk exerts its sleep-promoting effects through complex interactions with the gut-brain axis, primarily by modulating the intestinal microbiota and influencing neurotransmitter signaling. Dietary factors can alter neurotransmitter pathways, synaptic transmission, membrane fluidity, and signal transduction, thereby affecting multiple brain functions. In insomnia models, cow milk has been shown to significantly improve sleep parameters, including prolonged sleep time and reduced sleep latency. These effects are closely tied to changes in gut microbiota diversity, specifically, increased α-diversity and β-diversity, indicating a healthier and more balanced microbial environment. Milk consumption promoted the growth of beneficial bacteria such as *Bifidobacteria* and *Lactobacillus*, which are known to enhance sleep quality by reducing stress and contributing to neurotransmitter production. *Bifidobacteria* ferment lactose into lactic acid, inhibiting pathogenic bacteria, while *Lactobacillus* is positively correlated with improved sleep rhythm and quality. These probiotics can synthesize γ-aminobutyric acid (GABA), an inhibitory neurotransmitter involved in reducing central nervous system excitability. Additionally, the gut microbiota contributes to the synthesis of serotonin (5-HT), a key neurotransmitter in sleep regulation, through the metabolism of dietary tryptophan. This amino acid, absorbed in the small intestine, serves as a precursor to 5-HT, and its availability is influenced by both dietary intake and microbial synthesis. The neurotransmitters produced in the gut, such as GABA, 5-HT, glutamate (Glu), dopamine (DA), norepinephrine (NE), and histamine, interact with the central and autonomic nervous systems through pathways such as the vagus nerve, influencing hormonal secretion and the host’s overall physiological state. Furthermore, sleep deprivation is known to disrupt circadian gene expression and gut microbial structure, while milk intake has been shown to activate the CREB/BDNF signaling pathway in the brain, which is vital for regulating sleep cycles and neuronal plasticity [8].

Understanding the relationship between milk or dairy products and sleep requires more in-depth investigation, particularly regarding which specific components are related to sleep-promoting effects. While current evidence suggests that certain nutrients and bioactive compounds found in milk and dairy products, such as tryptophan, calcium, bioactive peptides, and melatonin, may play a role in sleep regulation, the mechanism remains unclear. Further studies are needed to understand how specific components of dairy products influence sleep and how they interact with physiological processes related to sleep. It is also essential to investigate the effects in specific populations, such as children, the elderly, or individuals with sleep disorders.

Given the high prevalence of sleep disorders among individuals, mainly women, studying non-pharmacological strategies to improve sleep is necessary. Sleep disorders not only impair daily functioning and clinical outcomes but also significantly reduce quality of life [3].

## Conclusions

This systematic review and meta-analysis of studies published between 1989 and 2025 evaluates the relationship between milk and dairy products and sleep. Some of the studies included were assessed as high quality, although two studies presented a high risk of bias, mainly due to the failure to blind the sample, the researchers, and the study population. The meta-analysis evaluation of data obtained from the scientific literature available about this systematic review demonstrated that the ingestion of milk and dairy products reduced the total sleep PSQI score in humans. Therefore, the evidence indicates that, according to the data available at the time of writing this work, regular consumption of milk and dairy products appears to have a beneficial effect on sleep quality.
